# CMIP: a software package capable of reconstructing genome-wide regulatory networks using gene expression data

**DOI:** 10.1186/s12859-016-1324-y

**Published:** 2016-12-23

**Authors:** Guangyong Zheng, Yaochen Xu, Xiujun Zhang, Zhi-Ping Liu, Zhuo Wang, Luonan Chen, Xin-Guang Zhu

**Affiliations:** 1 CAS Key Laboratory of Computational Biology and State Key Laboratory of Hybrid Rice, CAS-MPG Partner Institute for Computational Biology, Chinese Academy of Sciences, 320 Yueyang Road, Shanghai, 20031 China; 2 Software Engineering Institute, East China Normal University, 3663 North Zhongshan Road, Shanghai, 200062 China; 3 Key Laboratory of Systems Biology, Institute of Biochemistry and Cell Biology, Chinese Academy of Sciences, 320 Yueyang Road, Shanghai, 200031 China; 4 College of Life Science and Biotechnology, Shanghai Jiaotong University, 800 Dongchuan Road, Shanghai, 200240 China

**Keywords:** Gene regulatory network, Genome-wide, Parallel computing, Software

## Abstract

**Background:**

A gene regulatory network (GRN) represents interactions of genes inside a cell or tissue, in which vertexes and edges stand for genes and their regulatory interactions respectively. Reconstruction of gene regulatory networks, in particular, genome-scale networks, is essential for comparative exploration of different species and mechanistic investigation of biological processes. Currently, most of network inference methods are computationally intensive, which are usually effective for small-scale tasks (e.g., networks with a few hundred genes), but are difficult to construct GRNs at genome-scale.

**Results:**

Here, we present a software package for gene regulatory network reconstruction at a genomic level, in which gene interaction is measured by the conditional mutual information measurement using a parallel computing framework (so the package is named CMIP). The package is a greatly improved implementation of our previous PCA-CMI algorithm. In CMIP, we provide not only an automatic threshold determination method but also an effective parallel computing framework for network inference. Performance tests on benchmark datasets show that the accuracy of CMIP is comparable to most current network inference methods. Moreover, running tests on synthetic datasets demonstrate that CMIP can handle large datasets especially genome-wide datasets within an acceptable time period. In addition, successful application on a real genomic dataset confirms its practical applicability of the package.

**Conclusions:**

This new software package provides a powerful tool for genomic network reconstruction to biological community. The software can be accessed at http://www.picb.ac.cn/CMIP/.

**Electronic supplementary material:**

The online version of this article (doi:10.1186/s12859-016-1324-y) contains supplementary material, which is available to authorized users.

## Background

In the post-genome era, an important task of molecular biology is to reconstruct gene regulatory networks (GRNs), which represent interactions between genes inside a cell or tissue. A GRN provides molecular interactions and regulatory effects of components involved in a biological process, and hence provides insights into the molecular mechanism of the process [1, 2]. In detail, GRNs can be used to interpret biological processes through studying topological structure information of sub-networks related to these processes, where genes facilitate specific biological functions together [3, 4]. GRNs can help annotate genes clustered in modules and motifs since genes in the same module or motif have similar functions [5, 6]. GRNs can be utilized to identify dynamical network biomarkers (DNB) at the critical states of biological processes if stage-wise data are available, which help biologists understand mechanism of biological process better [7, 8]. Therefore, reconstruction of GRNs can not only support investigating roles of genes and components involved in a biological process, but also help study how a process is developed and maintained.

In the last decade, many algorithms have been developed to infer GRNs based on reverse-engineering methods, such as Bayesian network [9–11], Boolean network [12, 13], linear and non-linear regression [14–18], differential equation [19, 20], information-theoretic approaches [21–26], probabilistic phylogeny network [27], part mutual information network [28], and probabilistic graphical models [29–32]. In 2011, we proposed a GRN inference algorithm, named PCA-CMI, which can distinguish direct interactions of gene pairs from indirect ones based on the conditional mutual information (CMI) measurement [33–35]. However, two limitations of the algorithm hinder its wide application. One is that an appropriate threshold should be assigned to the method for direct interactions judgment in advance, which is difficult for users since the threshold is hard to select before GRN reconstruction. The other is that the method is time-costly especially for genomic network reconstruction, which is a common restriction of most current GRN inferring methods.

In this report, we describe a new software package CMIP, which implements the PCA-CMI algorithm with the goal of enable biologists to build genomic networks easily. The CMIP package incorporates a threshold determination method and a parallel computing process for network inference. The threshold determination method can choose an appropriate cutoff on-the-fly for gene interaction judgment. Computing procedure of the CMI measurement is optimized to make the algorithm robust, in which parallel computing strategies are applied to accelerate calculation process. This paper describes the algorithm details, program implementation, prediction performance, and practical application of the CMIP package.

## Methods

### Workflow of the CMIP package

**Fig. 1:**
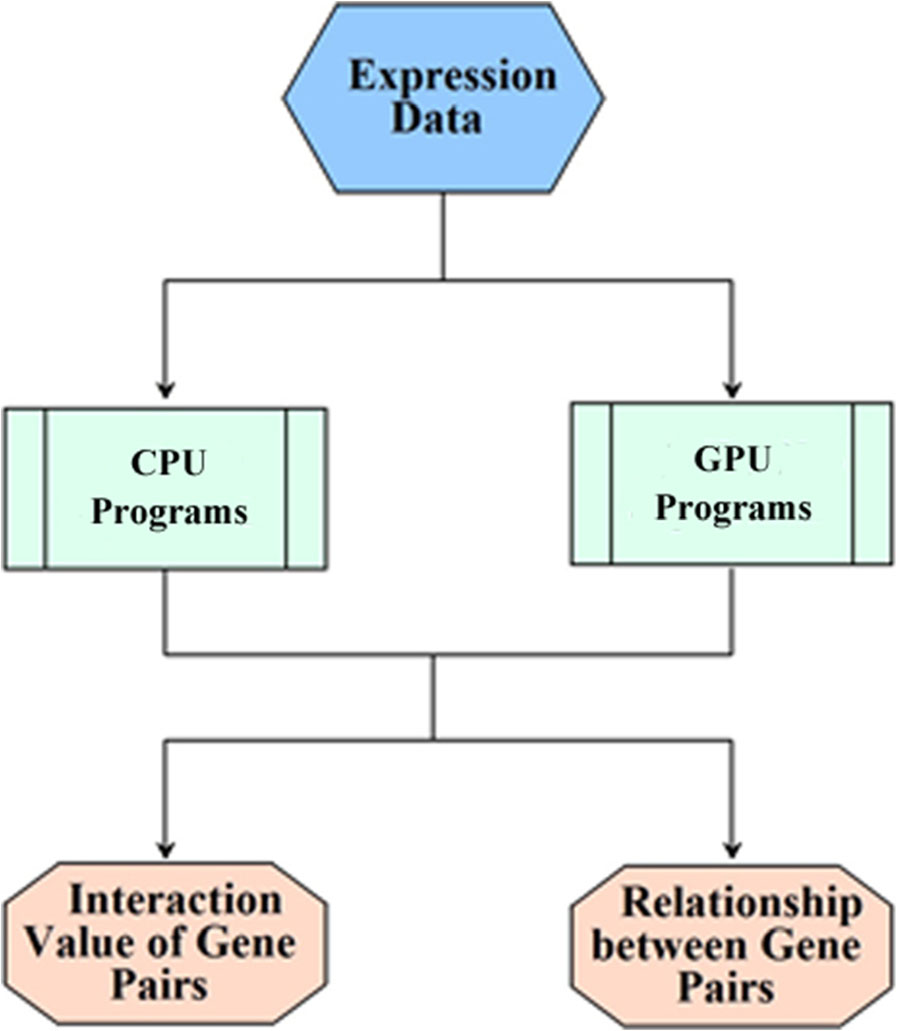
Workflow of the CMIP software package. First, expression datasets are used as input of the CMIP algorithm. Then the CPU or GPU programs are selected to reconstruct networks. Finally, result files recording interaction and relationship of gene pairs are generated as output

As showed in Fig. 1, the CMIP package first uses an expression data file as input, in which expression value of genes under different experimental conditions are provided. Then, CMIP program calculates the correlation value between gene pairs. During calculation process, a threshold determination method is called to generate an appropriate cutoff for direct interaction judgment. When the process is finished, two result files are produced as output. One is a gene interaction file, recording raw correlation value of gene pairs. The other is a gene relation file, providing the relationship between gene pairs. In practice, relationship of a gene pair is assigned as 1 when their correlation value is over the interaction cutoff. Otherwise corresponding value is assigned as 0.

### Correlation calculation of the CMIP algorithm

The algorithm implemented in the CMIP package is as follows. First, correlation values of each gene pairs are calculated using the mutual information (MI) measurement. Then a threshold determination method (described in the “Threshold determination of gene interaction” section) is called to provide an appropriate interaction cutoff for gene pairs. An interaction is marked for gene X and Y when their raw correlation value is over the cutoff. After that, for each gene interaction, their correlation values are updated through calculating the conditional mutual information (CMI) measurement (Eq. 1–4), which describes the dependence of two genes given neighboring genes as condition. A gene Z is defined as a neighbor of gene X and Y when it has interactions with both gene X and Y. In practice, the maximum CMI value between gene X and Y is kept. Finally, for gene X and Y, they are regarded as having direct interaction when their CMI value is over the interaction cutoff and their relationship value is set to be 1 as output.

1 $$I\left(X,Y\Big|Z\right)={\displaystyle \sum_{x\in X,y\in Y,z\in Z}p\left(x,y,z\right) log\frac{p\left(x,y\Big|z\right)}{p\left(x\Big|z\right)p\left(y\Big|z\right)}}$$

Where *I(X,Y|Z)* is CMI measurement between gene X and Y given gene Z as a condition; *p(x,y,z)* are joint probability of gene triple (X,Y,Z); while *p(x|z), p(y|z),* and *p(x,y|z)* are conditional probabilities of gene X, Y, and gene pair (X,Y) given gene Z as a condition. According to information theory, the CMI measurement can also be defined as follows.

2 $$H\left(X,Y\Big|Z\right)=H\left(X,Z\right)+H\left(Y,Z\right)-H(Z)-H\left(X,Y,Z\right)$$

Where *H(Z)* is the entropy of gene Z; *H(X,Z), H(Y,Z)* and *H(X,Y,Z)* are joint entropies of gene pair (X,Z), (Y,Z) and gene triple (X,Y,Z); *H(X,Y|Z)* is the conditional entropy of genes X and Y given gene Z as a condition. Based on the Gaussian distribution, the entropy of gene Z can be estimated as follows.

3 $$H(Z)= log\left[{\left(2\pi e\right)}^{n/2}{\left|C(Z)\right|}^{1/2}\right]=\frac{1}{2} log{\left(2\pi e\right)}^n\left|C(Z)\right|$$

Where n is number of experiment, *C(Z)* is the covariance matrix of gene Z, and |*C(Z)*| is the determinant of the matrix. While joint entropies can be estimated similarly through corresponding covariance matrixes. Based on the entropy estimator, in practice the CMI measurement (I) is calculated as follows.

4 $$I\left(X,Y\Big|Z\right)=\frac{1}{2} \log \frac{\left|C\left(X,Z\right)\left|\cdot \right|C\left(Y,Z\right)\right|}{\left|C(Z)\left|\cdot \right|C\left(X,Y,Z\right)\right|}$$

Where *C(X,Z)* and *C(Y,Z)* are covariance matrixes of gene pair (X,Z) and (Y,Z); *C(X,Y,Z) is* covariance matrix of gene triple (X,Y,Z); the |*C()*| is determinant of a matrix.

### Threshold determination of gene interaction

**Fig. 2:**
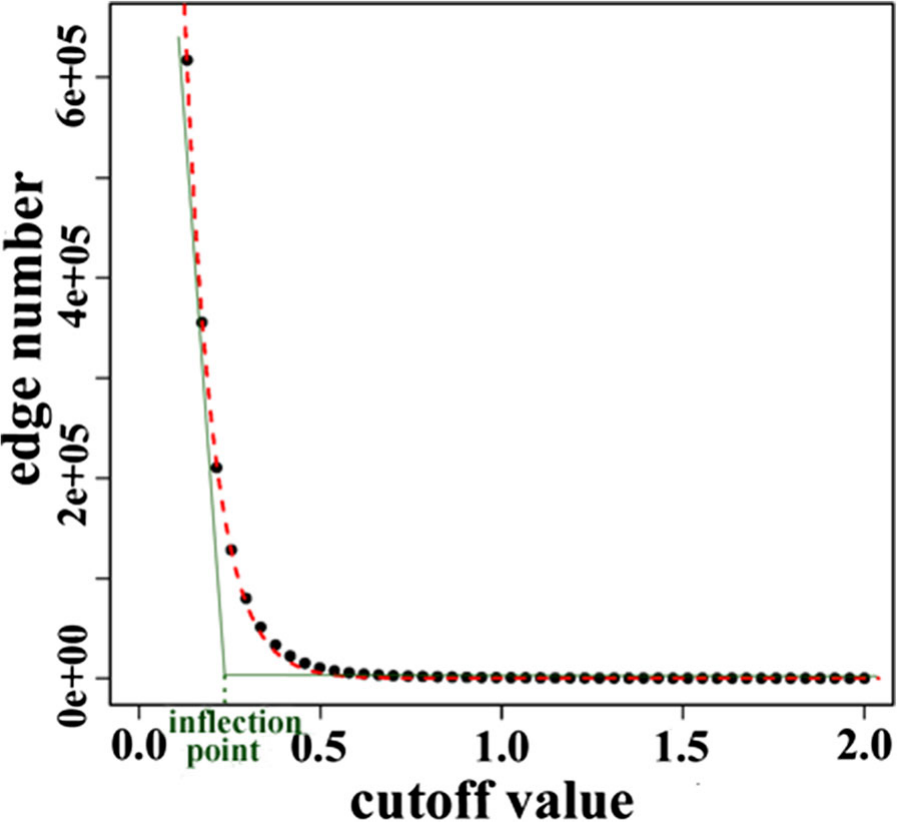
Diagram of threshold determination for gene interactions. Relationship between interaction and cutoff is first investigated, and then a fitting curve method based on exponential function is adopted to simulate relationship between them. Finally, the intersection of slope of the start and end sections of the fitting curve was chosen as the threshold

Given interaction of gene pairs, the number of interactions decreases dramatically with the increase of the cutoff and their relationship shows an exponential decay. Therefore, in practice we chose to use an exponential function to simulate relationship between interaction and cutoff. Correlation values of gene pairs are first calculated as mentioned in the “Correlation calculation of the CMIP algorithm” section. Then direct interactions between gene pairs under different cutoffs are estimated and a scatter plot is generated (Fig. 2), where X axis is the cutoff value and Y axis is the number of direct interactions. After that, we fit the number of direct interactions as a function of the cutoff value with an exponential function. Finally, we chose the threshold as the intersection of slope of the start and end sections of the fitting curve, which represents the inflection point of the curve.

### Parallelization of the CMIP programs

In CMIP, parallel strategies were applied to speed up computing process of correlation. In practice, a CPU and a GPU version program of CMIP algorithm were developed so that users could utilize them in different computational environment. The CPU version program is implemented based on the OpenMP framework [36], where loop calculation is accelerated with the multi-threads technology. In detail, the total computing task of correlation is first calculated based on gene numbers, and then computing tasks is partitioned equally to each CPU node. While the GPU version program is implemented based on the CUDA framework [37], where serial and parallel computing tasks are undertaken by CPU and GPU cores respectively. In detail, a production-consumption strategy is used in the GPU version program, in which gene expression data used by correlation calculation is first processed by the CPU cores (production); then pre-processed data is delivered to GPU cores for correlation calculation (consumption) using a parallel mode; finally, the results are transferred from GPU to CPU cores for aggregation.

### Evaluation of network inference methods

Receiver operating characteristic (ROC) curve and precision-recall (PR) curve are used to evaluate performance of different network inference methods. The ROC curve is a graphical plot which illustrates discrimination capacity of algorithm under various thresholds for binary classifier problems, where the X and Y axis are false and true positive rate respectively. While the PR curve shows recognition capability of algorithm under various thresholds for positive samples, in which the X and Y axis represent the recall and precision measurement respectively. Commonly, area under the ROC curve (AUROC) and area under the PR curve (AUPR) are calculated to comprehensively evaluate performance of a network inference method. In practice, the true positive rate (TPR, also known as recall), false positive rate (FPR), and positive predictive value (PPV, also known as precision) and accuracy (ACC) are calculated as follows.

5 $$\begin{array}{l}TPR=TP/\left(TP+FN\right)\\ {}FPR=FP/\left(TN+FP\right)\\ {}PPV=TP/\left(TP+FP\right)\\ {}ACC=\left(TP+TN\right)/\left(TP+FN+TN+FP\right)\end{array}$$

Where TP, TN, FP and FN are numbers of true positive, true negative, false positive and false negative respectively. Given a true interaction between genes X and Y, it is recorded as a true positive item if it is predicted by the algorithm. Otherwise, it is recorded as a false negative item. Similarly, for a non-interaction gene pair X and Y, it is recorded as a false positive item when predicted by the algorithm. Otherwise, it is recorded as a true negative item.

## Results and discussion

### Efficiency of the threshold determination method

In this study, we developed an automatic threshold determination method for interaction cutoff prediction. Here we show the efficiency of the method using numerical experiments. First, 10 benchmark datasets (synthetic datasets) were collected from the DREAM3 competition website [38]. Then, we ran CMIP programs on these datasets with 0-, 1-, 2-, or 3-order manner separately, which means the CMI is calculated given 0, 1, 2, or 3 neighboring genes as conditions. Note that when no neighboring gene is given as condition, the CMI measurement is equivalent to the MI measurement. In practice, the CMIP programs were run with a predefined cutoff, which was increased from 0 to 1 with a step size of 0.02. For each running of programs, accuracy of the CMIP algorithm under a certain cutoff was recorded. After that, accuracies under different cutoffs were checked, and the cutoff at which corresponding accuracy measure reached its maximum was stored as the true threshold. On the other hand, the CMIP programs were run with the automatic threshold determination method (see the “Threshold determination of gene interaction” section for details) and a predicted threshold was presented. Finally, offset of threshold was detected through comparing the true and predicted threshold values (Eq. 6).

6 $$offset=\frac{\left| true\ threshold- predicted\ threshold\right|}{maximum - minimum} \times 100\%$$

In our work, the stringent, standard, and moderate criteria are defined as offset less than 5, 10, and 20% respectively. Results of offset detection are shown in Table 1, where each cell represents the number of datasets for which the offsets satisfy a certain criteria under a defined order (0-, 1-, 2-, or 3-order). Totally, the proportion of datasets that satisfies the stringent, standard, and moderate criteria are 45, 75, and 93% respectively. These results demonstrate that the new threshold determination method developed in this study is effective and an appropriate cutoff can be provided on-the-fly during calculation process of correlation. Though in this study, we already include 10 datasets to test the threshold determination method, it is possible that there are networks for which the current threshold determination method might be not the best option; therefore, development of new methods for threshold determination is still needed in the future.

**Table 1:** Effectiveness of threshold determination method under different criteria

Criteria/manners	0-order	1-order	2-order	3-order	Percentage
Offset less than 5%	3	7	4	4	45%
Offset less than 10%	5	9	9	7	75%
Offset less than 20%	7	10	10	10	93%

### Parameter selection of the CMIP software

**Fig. 3:**
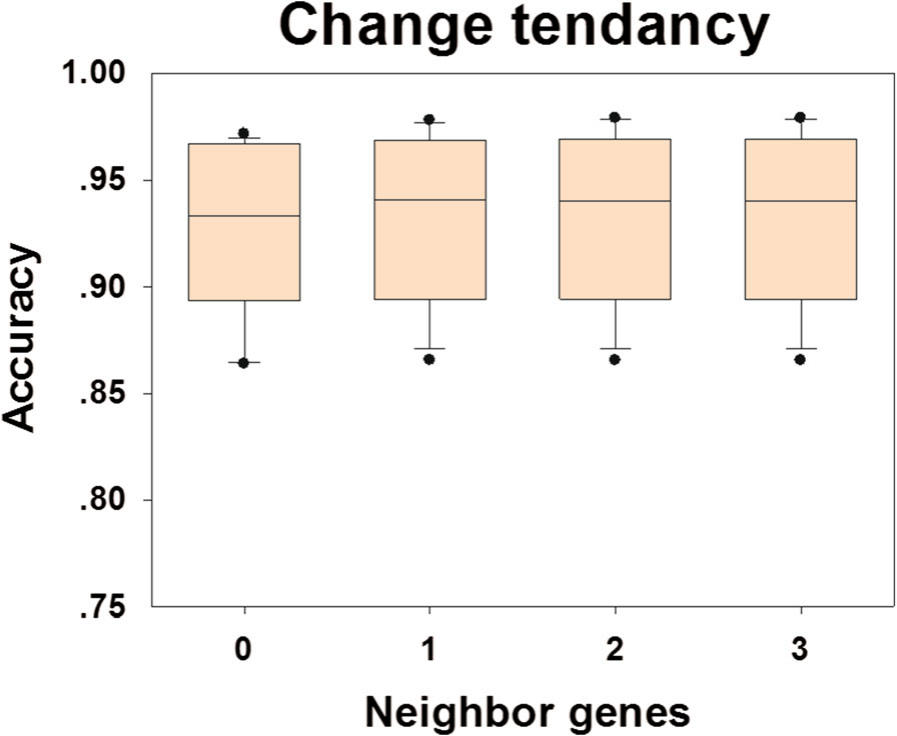
Changes of the accuracy measurement for the CMIP algorithm. The CMIP programs were conducted on 10 benchmark datasets with 0-,1-,2- and 3-order manners to test impacts of different order parameters

The CMIP algorithm is based on the CMI measurement, where computational complexity increases exponentially with the increase of the number of neighboring genes as conditions, i.e. with the increase of orders. So an appropriate order parameter needs to be selected for the algorithm. Here, we tested the impacts of different order parameters on prediction accuracy. In practice, 10 synthetic benchmark datasets were first collected from the DREAM3 website [38]. Then the CMIP programs were run on these datasets with 0-, 1-, 2-, and 3-order manners separately and the accuracy measurement was inspected in each running. Inspection results show that the accuracy of the CMIP algorithm gradually increased with the increase of the order parameter from 0 to 3; however, the increasing trend becomes flat after the order of 1 (Fig. 3). Considering both the accuracy and computational complexity, we recommend setting 1 to be the order parameter for the CMIP algorithm.

### Performance evaluation of the CMIP package on benchmark datasets

**Fig. 4:**
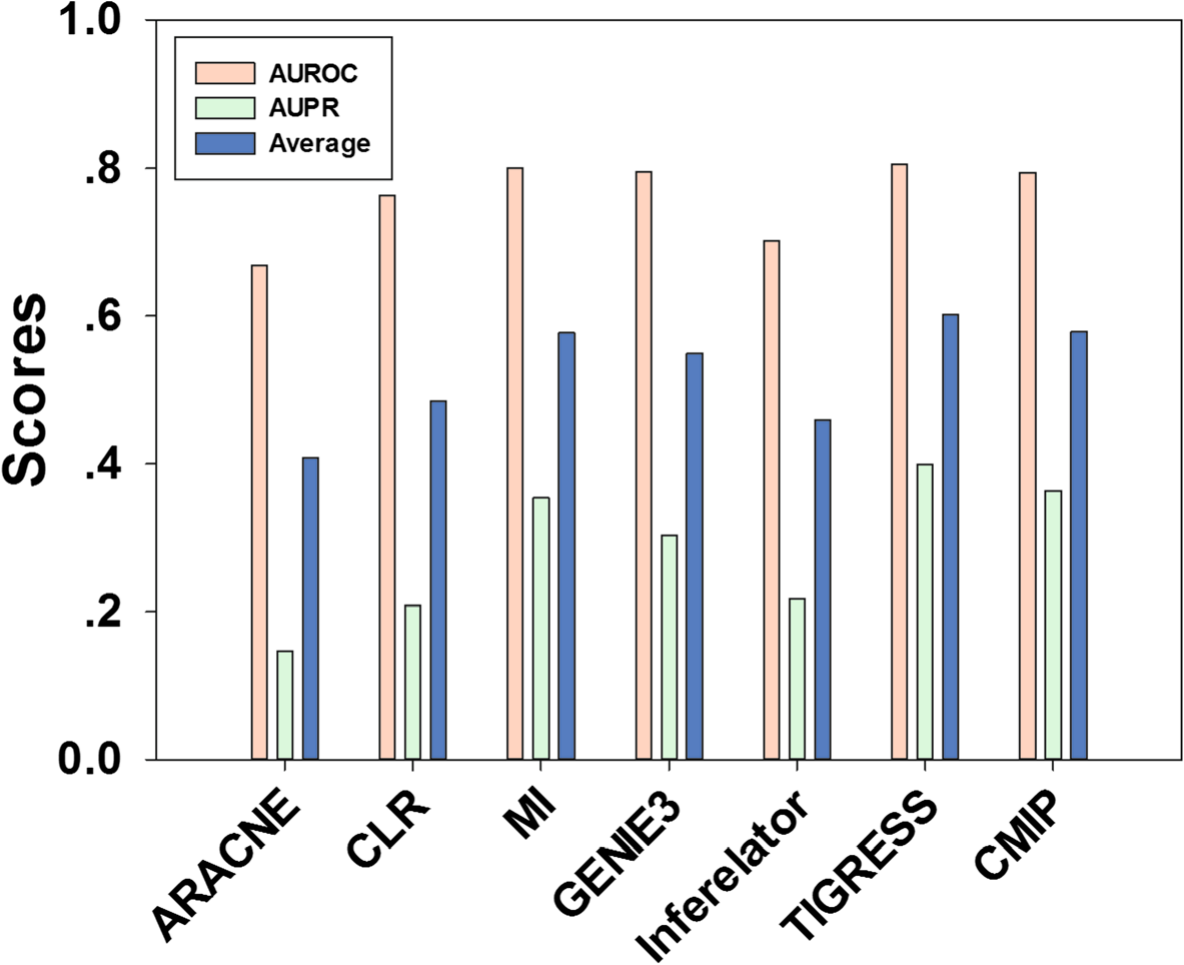
Scores of various network inference methods. Performance of various network inference methods are compared on 10 benchmark datasets using the AUROC and AUPR measurement

The CMIP package was utilized to reconstruct GRNs on 10 DREAM3 benchmark datasets for performance evaluation. In practice, programs of the package were run with a 1-order manner, i.e. the CMI measurement of gene pair was calculated given one neighbor gene as condition. Subsequently, running results of CMIP were compared with other popular network inference methods using the AUROC and AUPR measurements. Specifically, programs of popular GRN inference methods were downloaded from website of the DREAM projects [39] and recommended parameters were used during running these programs. Mean scores of the AUROC and AUPR measurement on 10 benchmark datasets for various methods are shown in Table 2 and Fig. 4. Given both the AUROC and AUPR measurements, the CMIP algorithm achieves high performance and delivers competitive values to popular methods. For average score of the AUROC and AUPR measurement, the CMIP algorithm performs better than all methods except the TIGRESS algorithm. These results demonstrate that the CMIP algorithm is comparable to most currently used network inference methods.

**Table 2:** Scores of various network inference methods on benchmark datasets

Measurements	ARACNE	CLR	MI	GENIE3	Inferelator	TIGRESS	CMIP
AUROC	0.6689	0.7632	0.8004	0.7955	0.7014	0.8048	0.7945
AUPR	0.1465	0.2076	0.3537	0.3033	0.2172	0.3991	0.3637
Average	0.4077	0.4854	0.5771	0.5494	0.4593	0.6020	0.5791

### Application of the CMIP package on real biological datasets

We further applied the CMIP software on real transcriptome data to check its practical applicability. The CMIP software was first used to build GRNs of pineapple leaves. In detail, a GRN of leaf base and a GRN of leaf tip were constructed based on genome-scale expression data. Totally, 15,483 genes (201,537 interactions) and 13,543 genes (188,391 interactions) were included in the base and tip GRNs respectively. Analysis of the node degree distribution suggested that both the tip and the base network showed small-world properties. Then, we extracted genes linked to metabolic enzymes of Crassulacean Acid Metabolism (CAM) in the base and tip networks. After that, genes linked to metabolic enzymes in the tip network but missed in the base network were identified as potential recruited regulators of CAM photosynthesis. Subsequently experimental study showed that regulators identified from network comparison do play important roles in photosynthesis differentiation [40]. This application of CMIP software on real dataset shows its effectiveness and efficiency for genomic GRNs reconstruction.

### Effectiveness of parallel computing framework of CMIP programs

Since the CMIP software is developed to infer GRNs for genome-scale datasets, parallel computation strategies are adopted in the software to speed up computing process. Here, we compared running time of the CMIP programs with popular network inference methods. In practice, CPU version programs were run on a Linux computing server, which has two Intel Xeon E5-2650 CPU units containing 32 CPU cores in total. While GPU version programs were run on a Linux computing server, which has 2 NVIDIA Tesla k20m GPU cards containing 4992 CUDA cores in total. In this study, all program was executed on a synthetic dataset (collected from published literatures), which included expression values of 500 genes under 343 biological treatments for Arabidopsis, Running times of different programs are shown in Table 3. Programs of the CMIP algorithm are much faster than most popular methods. Running time of the CMIP software applied in pineapple GRNs reconstruction is shown in Table 4. These results suggest that parallel computing strategies applied in the CMIP software are efficient and the software can handle genome-scale datasets within a reasonable time period.

**Table 3:** Running time of different network inference programs

Methods	CMIP^a^	CMIP^b^	MI	ARACNE	CLR	GENIE3	Inferelator	TIGRESS
Multi-threads	Yes	Yes	No	No	No	No	Yes	No
Time (seconds)	7	4	8	353	68	4715	2728	13089

^a^GPU version program, ^b^ CPU version program

**Table 4:** Running time of the CMIP programs in pineapple GRNs reconstruction

Programs/time (seconds)	Leaf base network	Leaf tip network
CMIP^a^	1828	2277
CMIP^b^	2026	2733

^a^GPU version program, ^b^ CPU version program

### Usage of the CMIP package

**Fig. 5:**
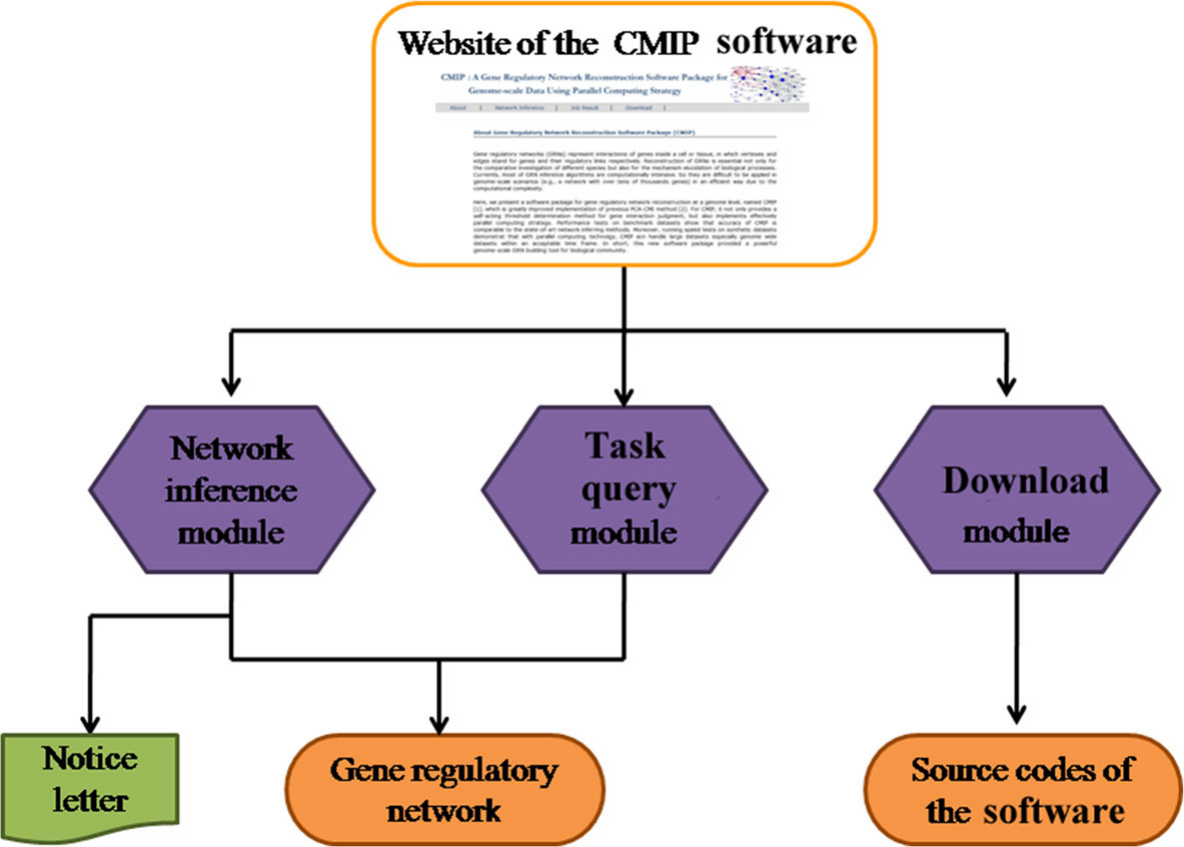
Data processing pipeline of website for the CMIP algorithm. First, users can input transcriptomics data and submit their computing tasks through the “Network Inference” module. When a task is finished, a notifying letter will be sent to users. Simultaneously, users can check results of their tasks on the “Task query” module. In addition, users can obtain the CMIP software in the “Download” module

A service website of the CMIP programs is established so that users can utilize them remotely (Fig. 5). Now, the website can be accessed at http://www.picb.ac.cn/CMIP/. The web service is created based on a remote resource management system. Once a task is submitted to the system, calculation resources include CPU and GPU computing components will be automatically assigned. To use the CMIP programs, users first need to submit their computing tasks through inputting expression data on the “Network inference” module. Then tasks are handled by the management system. When a task is finished, a notice letter will be sent to users. Alternatively, users can query status of their tasks through the “Job Result” module. Finally, summary information of the task will be presented on the website. In addition to the online manners, users can download the source codes of CMIP software from the “Download” module (Additional file 1), and then use it on local computing servers.

## Conclusions

In this study, we provide a new software package for network inference, which can reconstruct genomic GRNs within a short time period. The software package has a number of novel features compared with other GRN inference methods. First, CMIP can detect direct gene interactions from indirect ones with a high accuracy based on the CMI measurement. Results of performance evaluation on benchmark datasets show that precision and accuracy of the CMIP algorithms are comparable to most currently used methods. Secondly, an automatic threshold determination method is incorporated into the CMIP algorithm, so users do not need specify a predefined cutoff for gene interaction judgment and an appropriate threshold can be provided on-the-fly. Numerical experiments confirm the efficiency of the threshold determination method. Last but not least, the OpenMP and CUDA framework are applied in the software to speed up computing process of the CMIP algorithms, which enables the software to build GRNs with less running time. With this feature, the software is suitable to reconstruct genomic GRNs. The area of CMIP that needs future development is that it can’t provide directionality to edges of gene regulatory networks, which is a common limitation of many current methods, such as CLR [24] and minet [22]. This limitation can be resolved by a two-steps routine. First, using the CMIP software to build a gene regulatory network as background model, then giving directionality to edges of the network according to results of biochemical perturbation experiments, or predicting directionality for edges of the network based on time series expression data [41].

## Additional file

Additional file 1: S1- CMIP_code.zip: source codes of the CMIP software package. (ZIP 46 kb)
